# A personalized intervention to prevent depression in primary care: cost-effectiveness study nested into a clustered randomized trial

**DOI:** 10.1186/s12916-018-1005-y

**Published:** 2018-02-23

**Authors:** Anna Fernández, Juan M. Mendive, Sonia Conejo-Cerón, Patricia Moreno-Peral, Michael King, Irwin Nazareth, Carlos Martín-Pérez, Carmen Fernández-Alonso, Antonina Rodríguez-Bayón, Jose Maria Aiarzaguena, Carmen Montón-Franco, Antoni Serrano-Blanco, Inmaculada Ibañez-Casas, Emiliano Rodríguez-Sánchez, Luis Salvador-Carulla, Paola Bully Garay, María Isabel Ballesta-Rodríguez, Pilar LaFuente, María del Mar Muñoz-García, Pilar Mínguez-Gonzalo, Luz Araujo, Diego Palao, María Cruz Gómez, Fernando Zubiaga, Desirée Navas-Campaña, Jose Manuel Aranda-Regules, Alberto Rodriguez-Morejón, Juan de Dios Luna, Juan Ángel Bellón

**Affiliations:** 1 ParcSanitariSant Joan de Déu, FundacióSant Joan de Déu, C/Dr. AntoniPujadas, 42, 08830 SantBoi de Llobregat, Barcelona Spain; 2 Mental Health Policy Unit, Brain and Mind Centre, Faculty of Health Sciences, University of Sydney, Sydney, Australia; 3 Centro de Salud La Mina, C/Mar s/n, 08930 Barcelona, Spain; 4 Distrito de AtenciónPrimariaMálaga-Guadalhorce, Unidad de Investigación, C/Sevilla, 23, 3a Planta, 29009 Málaga, Spain; 5 Division of Psychiatry, University College London, Charles Bell House, 67-73 Riding House Street, London, W1W 7EH UK; 6 Department of Primary Care & Population Health, University College London, Royal Free Site, Rowland Hill Street, London, NW3 UK; 7 Centro de SaludMarquesado, Distrito Sanitario Granada Nordeste, Avenida Mariana Pineda s/n, 18500 Granada, Spain; 8 Gerencia Regional de Salud de Castilla y León, Paseo de Zorrilla, 1, 47007 Valladolid, Spain; 9 Centro de Salud San José, Plaza JuanfraGarridoRomera s/n, 23700 Linares, Jaén Spain; 10 Centro de Salud San Ignacio, LarrakotorreKalea, 9, 48015 Bilbao, Bizkaia Spain; 11 Centro de Salud Casablanca, C/Viñedo Viejo, 10, 50009 Zaragoza, Spain; 12 ParcSanitariSant Joan de Déu, C/Dr. AntoniPujadas, 42, 08830 SantBoi de Llobregat, Barcelona Spain; 13 Centro de InvestigaciónBiomédica en Red de Salud Mental de la Universidad de Granada, Facultad de Medicina PTS Avda.de la Investigación (Departamento de Psiquiatría Torre A Planta 9a), 11, 18016 Granada, Spain; 14 Centro de Salud La Alamedilla, UnidadInvestigación, AvenidaComuneros 27-31, 37003 Salamanca, Spain; 15 Centre for Mental Health Research.Research School of Population Health, ANU College of Health and Medicine-Australian National University, 63 Eggleston Rd, Acton, ACT 2601 Australia; 16 Unidad de Investigación de AtenciónPrimaria, C/Luis Power, 18-4o Planta, 48014 Bilbao, Spain; 17 Centro de Salud Federico del Castillo, C/Ramón Espantaleón s/n, 23005 Jaén, Spain; 18 Centro de Salud Andorra, C/de Huesca, 0, 44500 Teruel, Spain; 19 Gerencia Regional de Salud de Castilla y León, Unidad de Investigación, Paseo de Zorrilla, 1, 47007 Valladolid, Spain; 20 Hospital UniversitariParcTaulí, Servei de Salut Mental, ParcTaulí, 1, 08208 Sabadell, UniversitatAutònoma de Barcelona. CIBERSAM, Barcelona, Spain; 21 Centro de SaludArrabal, Unidad de Investigación de AtenciónPrimaria, AndadorAragues Puerto, 2-4, 50015 Zaragoza, Spain; 22 Centro San Andrés-Torcal, C/José Palanca, 29003 Málaga, Spain; 23 Departamento de Personalidad, Evaluación y TratamientoPsicologico de la Facultad de Psicologia de la Universidad de Málaga, Campus Teatinos s/n, 29590 Málaga, Spain; 24 Departamento de Bioestadística, Facultad de Medicina, Universidad de Granada ParqueTecnológico de Ciencias de la Salud, Avda de la Investigación 11, 18016 Granada, Spain; 25 Centro de Salud El Palo, Departamento de MedicinaPreventiva y Psiquiatría, Universidad de Málaga, Malaga, Spain; 26 Consorcio de Investigación Biomédica en Red de Epidemiología y Salud Pública, CIBERESP, Madrid, Spain

**Keywords:** Depression, Risk assessment, Cost-effectiveness

## Abstract

**Background:**

Depression is viewed as a major and increasing public health issue, as it causes high distress in the people experiencing it and considerable financial costs to society. Efforts are being made to reduce this burden by preventing depression. A critical component of this strategy is the ability to assess the individual level and profile of risk for the development of major depression. This paper presents the cost-effectiveness of a personalized intervention based on the risk of developing depression carried out in primary care, compared with usual care.

**Methods:**

Cost-effectiveness analyses are nested within a multicentre, clustered, randomized controlled trial of a personalized intervention to prevent depression. The study was carried out in 70 primary care centres from seven cities in Spain. Two general practitioners (GPs) were randomly sampled from those prepared to participate in each centre (i.e. 140 GPs), and 3326 participants consented and were eligible to participate. The intervention included the GP communicating to the patient his/her individual risk for depression and personal risk factors and the construction by both GPs and patients of a psychosocial programme tailored to prevent depression. In addition, GPs carried out measures to activate and empower the patients, who also received a leaflet about preventing depression. GPs were trained in a 10- to 15-h workshop. Costs were measured from a societal and National Health care perspective. Qualityadjustedlife years were assessed using the EuroQOL five dimensions questionnaire. The time horizon was 18 months.

**Results:**

With a willingness-to-pay threshold of €10,000 (£8568) the probability of cost-effectiveness oscillated from 83% (societal perspective) to 89% (health perspective). If the threshold was increased to €30,000 (£25,704), the probability of being considered cost-effective was 94% (societal perspective) and 96%, respectively (health perspective). The sensitivity analysis confirmed these results.

**Conclusions:**

Compared with usual care, an intervention based on personal predictors of risk of depression implemented by GPs is a cost-effective strategy to prevent depression. This type of personalized intervention in primary care should be further developed and evaluated.

**Trial registration:**

ClinicalTrials.gov, NCT01151982. Registered on June 29, 2010

## Background

In Western societies depression is viewed as a major and increasing public health issue, as it causes high levels of distress for those who experience it and their relatives, as well as considerable financial costs to society. Indeed, in 2013 major depression ranked fourth in the top ten causes of years lived with disability in Europe [1], with an estimated economic burden of €113.4 billion, explained by the use of services, losses in productivity and premature death due to suicide [2].

In an effort to reduce this burden, governments have supported increases in clinical services for mental disorders. However, despite this investment, the prevalence of depression has not changed. In community-representative studies major depression reached an incidence rate that is high (3.0%) relative to the number of prevalent cases (4.7%) [3]; therefore, it will be very difficult to reduce the prevalence unless the incidence is also reduced, and this is only possible by primary prevention. In addition, evidence suggests that although effective treatments for depression are available, they can reduce the burden by only 20% [4] because not all cases are recognized and not all people with recognized depression are treated and adhere to treatment.

Different studies have shown that depression is preventable [5–7]. In addition, prevention of depression is relatively good value for money [8]. However, the effect sizes of prevention are small, and most of the interventions are implemented by mental health specialists [5–7]. This jeopardizes its translation to primary health care centres, which may be a good setting for implementing preventive interventions [9]. Primary prevention aims to avoid the occurrence of disease by either eliminating the risk factors or increasing resistance to disease, so its application requires that its target population does not have the disease (depression in our case). Classically, primary prevention of depression is classified as ‘universal’ when it is applied to the general population, ‘selective’ to participants with some risk factor(s) for depression and ‘indicated’ to patients with subthreshold depression (they have some symptoms of depression but do not meet the criteria for diagnosis).The best primary prevention programme is likely to be one which targets modifiable risk factors, empowers individuals to address their risks and is inexpensive and capable of large-scale dissemination [10]. The PredictDalgorithm [11, 12] provides a quantification of major depression risk as well as information on risk factors for each individual that could guide prevention. We have recently evaluated the effectiveness of this strategy: compared with usual care, this new preventive intervention reduced the incidence of depression by more than 20% at 18 months [13]. This has been the first trial evaluating the effectiveness of a preventive intervention for depression based on the level and profile of risk and conducted by general practitioners (GPs). In this paper we present the results of the cost-effectiveness analysis of this intervention.

## Methods

### The PredictD trial

Full details of the PredictD protocol and effectiveness analysis are available elsewhere [13, 14]. Briefly, the PredictD-Cluster, Controlled, Randomized Trial (CCRT) was a national, multicentre, randomized controlled trial with two parallel arms, cluster assignment by primary care centre and 18 months of follow-up (from October 2010 to July 2012).

A total of 220 primary care centres from seven Spanish cities (Barcelona, Bilbao, Granada, Jaen, Málaga, Valladolid and Zaragoza) were approached. We conducted meetings in each centre to explain the project and invite physicians to participate; 118 (53.64%) out of the 220 centres were interested in participating. Seventy centres (10 per city) out of the 118 were randomly selected. A total of 193 physicians from the 70 centres consented to participate. Of those who accepted, we randomly selected two physicians per centre (i.e. 140 physicians). Random selection was conducted using sealed opaque envelopes by an independent researcher who was not part of the research team. Randomization to intervention or control group was conducted at the centre level. In each city five centres were assigned to the control group and five centres to the intervention group.

Research assistants randomly selected four to six patients per day from the patients with an appointment with the GP, using random starting points for each day, generated using a random number generator. GPs reviewed the list each day, excluding those patients who were not eligible for the trial. A total of 8292 participants were selected. Of these, 3056 were excluded in this first stage for the following reasons: 1479 were < 18 or > 75 years old; 1039 attended the surgery on behalf of the person who had the appointment; 153 would be away (> 4 months) during the follow-up; 122 had a documented severe mental disorder; 121 did not speak or understand Spanish; 88 had cognitive impairment; 54 had terminal illnesses. The process ended when there were 26–27 eligible patients for each GP.

A total of 5236 persons were invited to participate in the study by the research assistants. Of these, 1453 patients (27.28%) declined to participate. When compared with participants, these non-participants were slightly more likely to be male (38.4% versus 36.5%) but were of similar age (50.5 versus 50.7 years).

The 3783 patients who agreed to participate were then interviewed to detect major depression using the Composite International Diagnostic Interview (CIDI). Of the 3783 patients,457 (12.08%) met criteria for major depression in the last 6 months and were consequently excluded.

A total of 3326 patients (1663 in each arm), nested in 140 GPs from 70 primary care centres, composed the sample of the trial. The total number of patients with missing data in any of the outcome variables at any point was 577 (17.35%).

Although patients did not consent to randomization, patients at the intervention centre consented to receive the intervention, and all patients agreed to data collection. Neither the patients nor the GPs were blind to the intervention, which is common for trials that evaluate psychosocial interventions [15].The interviewers who assessed outcomes, however, were masked regarding allocation to study group. Local Ethics and Human Research Committees at each city approved the protocol.

### The Spanish primary care context

The National Health System of Spain provides universal coverage for citizens and foreign nationals (including undocumented immigrants). It is funded through taxes and free at the point of contact. Health care services are distributed into Health Areas and Basic Health Zones according to geographical, epidemiological and socio-economic criteria. Each Health Area covers a population of 200,000–400,000 inhabitants and is composed of several Basic Health Zones, which are the minimum units of health care organization. Basic Health Zones are organized around a primary care centre covering 5000–35,000 inhabitants. The primary care teams are composed of GPs, paediatricians, nurses and, in some cases, social workers. They provide a broad range of services, including the treatment of common mental disorders (shared with mental health specialists in severe cases) such as anxiety or depression [16], health promotion and preventive services. All the primary care centre staff members, including the GPs, are salaried. GP salaries contain two elements: a larger fixed payment and a smaller incentive, based on elements such as numbers of patients assigned, fulfilment of objectives, patterns of prescription and pay-for-performanceincentives [17].

### Interventions

#### Intervention group

The PredictD intervention has been described in detail elsewhere [14]. Briefly, the intervention started with the physician receiving the patient’s risk factors for depression and overall probability of developing depression in the next 12 months, using the Spanish version of the PredictD algorithm [11]. The PredictD algorithm is composed of 12 risk factors: six are patient characteristics or past events (sex, age, sex*age interaction, education, childhood physical abuse, probable lifetime depression), and six refer to current status (Short Form Health Survey (SF-12) physical score, SF-12 mental score, dissatisfaction with unpaid work, number of serious problems in very close persons, dissatisfaction with living together at home, taking medication for stress, anxiety or depression). The PredictD algorithm provides, in addition to the quantification of the overall risk of depression, knowledge of those risk factors influencing a given patient and that could guide a possible preventive intervention. Once the risk was calculated, the GP communicated this risk to the patient, and they worked together on a plan to manage those individual risk factors. This plan was tailored to the patients following a bio-pyscho-family-social framework, emphasizing measures to empower and activate the patients. In addition, all patients received a patient-oriented booklet on preventing depression, based on basic recommendations for self-care, including advice on exercise and sleep. All the GPs in the intervention arm were trained in a 10- to 15-h workshop on the prevention of depression using the PredictD risk algorithm.

#### Control group

GPs in the control arm did not receive the training or any information on their patients’ risk factors for depression or their probability of developing depression. They were simply asked to treat their patients as usual.

### Economic analysis

The economic evaluation was conducted from two perspectives: (1) societal perspective, including the costs of all types of health services (direct costs) and the costs that stem from production losses (indirect costs), and (2) a National Health System perspective (including only direct costs from the Spanish public health services). The time frame of this study was 18 months. Therefore, we discounted both costs and effects at 3.5% following National Institute for Health and Care Excellence (NICE) recommendations [18]. All costs were expressed in euros (€) for the reference year 2012.

#### Cost

We used a modified version of the Client Service Receipt Inventory (CSRI) [19] to collect information about use of health care resources, use of psychotropic drugs (antidepressants, anxiolytics and sedative-hypnotics) and lost productivity.

Direct health costs were calculated by multiplying the number of health service contacts/units (consultations, hospital days, etc.) by their standard cost price. This unit cost was retrieved from ‘Oblikue dataset (esalud)’ (http://www.oblikue.com/), which includes the official health services tariffs of the different Spanish autonomous communities. Cost of medication was calculated by multiplying cost price per daily dose, multiplied by the number of prescription days recommended, as recalled by the patient. Information about medication costs was obtained from the Spanish Pharmaceutical Vademecum (http://www.vademecum.es/). Indirect costs consisted of the costs of being on sick leave from paid work. Costs of work loss were calculated by multiplying the days on sick leave by the minimum daily wage in Spain according to the human capital approach. In addition, self-reported presenteeism was assessed using some questions from the World Health Organization (WHO) Health and Work Performance Questionnaire (HPQ) [20]. For this assessment, respondents first estimated how many days during the past 4 weeks they had been at work not being able to perform their job as usual (A), and then, they rated their overall work performance during these days using a 0–100 scale where 0 corresponds to doing no work at all (while at work) and 100 signifies top work performance (B). A score was calculated as follows: ((100 – B)*A)*6. Here 6 is the period of the follow-up (6 months).

Intervention costs included the cost of the booklet (€0.16 per patient) and the cost associated with the training of the physicians. Training was included in the GPs’ general training programme during working time, so no extra hours were worked or locums needed. No charges were incurred for training venues, as the training was conducted in the health centres or other health sector free venues. We included the cost of the trainer (€100 per h), estimated at 10 h and 7 groups (€7000), and the cost of the 70 dossiers delivered to the GPs with the basic information (€700). The intervention was embedded in the current practice. Participants in the intervention group were required to meet at least three times during the intervention (at baselineand at 6 and 12 months): in each of the three GP-patient interviews the GP communicated to the patient specific and updated information on his/her risk of depression, and then the patient and GP worked on a personalized plan for prevention of depression. These visits lasted approximately from 5 to 15 min, and this time generally was proportional to the level of risk. If the GP considered that the complexity of the case would require more visits, it was proposed to the patient. The patient at his/her own request could also propose new visits to the GP. All visits that were made during the follow-up, both compulsory and optional, were taken into account for costs.

The unit costs used are given in Table 1.

**Table 1:** Unadjusted costs and effects, by group

Costs	Unit cost (€2012)	Mean costs, control (95% CI)	Mean costs,intervention (95% CI)	Unadjusted diff (95%CI)^a^	*P* value
Costs associated to the intervention		€0	€4.79	NA	NA
Primary care physician	€10.50 (centre visit),€27.48 (home visit)	€96.61 (90.54 to 102.68)	€91.46 (85.68 to 97.23)	€–5.16 (–13.58 to 3.27)	0.230
Primary care nurse	€10.03 (centre visit),€25.37 (home visit)	€35.67 (28.74 to 42.59)	€26.34 (21.23 to 31.45)	€–9.32 * (–17.95 to –0.69)	0.032
Social worker	€14.90 (centre visit),€25.37 (home visit)	€0.63 (0.41 to 0.87)	€0.46 (0.27 to 0.66)	€–0.18 (–0.51 to 15)	0.293
Emergency visits to primary care	€63.99	€33.07 (25.27 to 40.86)	€29.05 (22.20 to 35.90)	€–4.02 (–14.44 to 6.40)	0.451
Total primary care		€162.75 (151.01 to 174.49)	€146.17 (135.76 to 156.58)	€–16.58 (–32.32 to –0.85)	0.039
Outpatient mental health (MH) visit	€42.93 (psychiatrist),€70.61 (psychologist),€10.03 (MH nurse),€19.22 (group therapy)	€16.16 (9.74 to 22.57)	€16.31 (8.08 to 24.54)	€0.15 (–10.31 to 10.62)	0.997
Antidepressant	*Different values*	€20.70 (0 to 72.8)	€22.32 (0 to 76.77)	€1.62 (–57.74 to 60.98)	0.658
Admission to psych.hospital^b^	€284.90	NA	NA	NA	NA
Total mental health		€45.19 (31.02 to 59.36)	€34.40 (24.07 to 44.73)	€–10.79 (–27.11 to 5.54)	0.184
Other outpatient specialists	€51.08	€149.08 (122.96 to 175.19)	€149.46 (122.53 to 176.40)	€0.38 (–35.26 to 36.02)	0.973
Diagnostic tests	*Different values*	€212.86 (189.47 to 236.25)	€198.24 (176.59 to 219.89)	€–14.61 (–46.31 to 17.08)	0.368
Emergency visits to hospital	€155.50	€96.18 (73.13 to 119.24)	€92.82 (70.60 to 115.04)	€–3.37 (–34.20 to 27.47)	0.831
Non-mental health-related admissions	*Different values*	€493 (134.79 to 852.27)	€548.04 (0 to 1136.96)	€54.51 (–141.48 to 250.50)	0.587
Total healthcare direct costs		€1039.11 (819.24 to 1258.98)	€1075.11 (849.70 to 1300.53)	€36.01 (–132.02 to 204.04)	0.676
Sick leave	€21.11/day	€337.37 (122.08 to 552.68)	€335.30 (96.60 to 574.03)	€–2.08 (–126.1 to 121.94)	0.974
Total costs		€1353.99 (1039.31 to 1668.66)	€1394.83 (1020.07 to 1769.6)	€40.85 (–178.21 to 259.91)	0.716
Units for sensitivity analysis					
Extra intervention costs^c^		**0**	€31.72	NA	NA
Private direct health costs	*Different values*	€174.13 (76.92 to 271.33)	€126.00 (82.90 to 169.10)	€–48.13 (–107.10 to 10.83)	0.099
Absenteeism	€21.11/day	€87.34 (57.27 to 117.42)	€89.70 (63.38 to 116.01)	€2.35 (–30.66 to 35.37)	0.888
Presenteeism	€21.11/day	€80.41 (36.78 to 124.04)	€63.39 (34.42 to 92.34)	€–17.02 (–52.93 to 18.81)	0.322
Effects	Unit of the effect	Effect gained control (95% CI)	Effect gained intervention (95%CI)	Unadjusted diff (95%CI)^a^	*P* value
QALYs	Mean	1.22 (1.20 to 1.24)	1.25 (1.23 to 1.26)	0.03 (–0.02 to 0.07)	0.240
QALYs-VAS	Mean	1.05 (1.04 to 1.07)	1.08 (1.07 to 1.10)	0.03 (0.006 to 0.05)*	0.014

^a^The means of the costs by group and the unadjusted difference have been calculated using univariatemaximum likelihood (ML)generalized linear model (GLM) family gamma and link log (cost as dependent variable and group as the only independent variable) and the command margins in 20 imputed databases. As it is not a linear model, the sum of the individual components of the costs  may be slightly different to the total cost presented

^b^There are only 8 participants who were admitted in an inpatient psychiatric unit, 7 in the control group and 1 in the intervention group. The length of stay has a mean of 10 days

^c^Extra costs related to the intervention if hiring of the venue and additional hours of the physician were included

*CI* confidence interval,*NA* not applicable,*QALY* quality-adjusted life year, *VAS* visual analogue scale

#### Health effects

Quality-adjusted life years (QALYs) were measured using the EuroQol five dimensions questionnaire (EQ-5D). The EQ-5D instrument has two parts. Part 1 is a self-reported description of health problems according to a five-dimensional classification (mobility, self-care, usual activities, pain/discomfort and anxiety/depression). Patients mark one of three levels of severity (1 = no problems, 2 = some/moderate problems and 3 = severe/extreme problems) in each dimension. Combinations of these categories define a total of 243 different health states. For instance, perfect health is coded as ’11111’. Each one of these health states has a ’weight’ or ’utility’ based on community preferences (i.e. social tariffs), where 1 represents perfect health, 0 death and negative numbers symbolize health states that are considered worse than death. Spanish social tariffs were used to estimate the utility of health states described by patients [21]. QALYs were calculated by multiplying the utility by the amount of time a patient spent in a particular health state. Linear interpolation was used for transitions between health states at baseline and at 6, 12 and 18 months. Part 2 is avisual analogue scale (VAS), graded from 0 (worst imaginable health status) to 100 (best imaginable health status), which is used by patients to estimate the ’value’ of their health status. We transformed the VAS to a scale from 0 to 1 and used it to have a ’proxy’ of an ’individual tariff’ and to calculate QALYs using it (referred to in the results as QALYs-VAS) [22].

#### Statistical analysis

Analyses were performed based on the intention-to-treat (ITT) principle, analysing all participants according to their randomized treatment and using multiple imputations when outcomes were missing. Incremental cost-effectiveness ratios (ICERs) were calculated as the difference in the cost between the intervention and the control group, divided by the difference in QALYs. The incremental costs and incremental health effects were modelled by generalized linear models (GLMs). We calculated the intraclass correlation coefficients (ICCs) of the health centre, GP and both, taking the costs and QALYs as dependent variables. The ICCs for the health centre were significant for the effect, while the ICC for the GP was significant for the costs. Thus, we used multilevel GLMs to account for such clustering effects. GLMs were fitted using different distribution families (Gaussian, inverse Gaussian, Poisson and gamma) and link functions (identity and log). Mmodified Park tests were used to select the appropriate family. To identify the correct link function, we compared the model performance of all permutations of candidate link and variance function using different diagnostic tests [23]. The best solution for costs was obtained using a gamma family and a log link. For QALYs, the most adequate family was Gaussian with an identity link.

All models were adjusted by their respective baseline values (i.e. QALYs or cost), the individual risk of depression (i.e. risk score from the PredictD algorithm) and the following variables,which were unbalanced at baseline (and were not included in the PredictD algorithm): employment status, owner/occupier accommodation, perception of safety inside/outside the home, anxiety disorder, experiences of discrimination, city.

We accounted for missing outcomes by using multiple imputations with chained equations under a missing at random (MAR) framework. We generated 50 imputed samples. Estimates for the descriptive analysis were combined using Rubin’s rules [24].

The analytic focus on cost-effectiveness (or cost-utility analysis) emphasizes the estimation of the joint density of cost and effects differences, the quantification of uncertainty surrounding the ICER and the presentation cost-effectiveness acceptability curves (CEACs). In that sense, in economic evaluations it is considered inappropriate to carry out separate and sequential hypothesis tests on differences in effects and costs to determine if incremental cost-effectiveness should be estimated (i.e. hypothesis testing is not conducted, so *P* values are not taken into consideration [25].

To deal with uncertainty, non-parametric bootstraps were used to simulate 1000 ICERs per imputed database (i.e. 50,000 ICERs in total), which were plotted on the cost-effectiveness plane. CEACs were then constructed. Each CEAC was derived from the net benefit approach:

$$ \mathrm{Net}\;\mathrm{monetary}\kern0.17em \mathrm{benefit}=\uplambda \times \left(\varDelta\;\mathrm{Effect}\right)-\left(\varDelta\;\mathrm{Cost}\right), $$

where λ represents the amount of money society is willing to pay to gain one extra unit of effect. All bootstrapped pairs of ∆ Effect and ∆ Cost (i.e. 50,000) were used to calculate the CEACs. Willingness-to-pay values ranged from €0 to 100,000 [26]. We have selected as optimal threshold of €30,000 per QALY ($32,058, £25,704), following Spanish suggestions [27]. This threshold fits into the cost-effectiveness threshold ranging between £20,000 and 30,000 used by NICE [18]. However, it is lower than the $50,000 suggested in the USA [28].

#### Sensitivity analyses

A number of sensitivity analyses were conducted in order to assess the robustness of the results:

1. Modifying the perspective of analysis, i.e. including only costs related to the outcome (primary health services, mental health services and psychotropic) and all the possible costs,i.e. private costs, absenteeism and presenteeism, and potential intervention-related costs including costs of hiring the venue (€100 per day, per 2 days in 7 cities) plus the costs associated with the time for which the physician attended the course (€10.5 per visit per 60 visits in two days per 70 physicians)
2. Modifying the discount rate, both in costs and effects, from 0 to 6%, following NICE recommendations [18]
3. Modifying the unit costs by doubling and halving them
4. Modifying the statistical analyses— using seemingly unrelated regressions (SURs), a method that consists of a system of regression equations that recognize the correlation between individual costs and outcomes [29], a completers approach (applying inverse probability weighting to address attrition bias) and models adjusted only by cost or QALYs at baseline

## Results

### Participants

The participants in the two groups were similar with regard to gender (63.6% female in the control group and 63.5% in the intervention group), age (51.5 and 50 years in the control and intervention groups, respectively), marital status (68.4% and 69.9% were married in the control and intervention groups, respectively) and educational level (42.2% and 44.3% had primary level education, respectively). However, they differed in key aspects related to the trial. Participants in the intervention group had a higher risk of depression,a slightly worse mental health-related quality of life, more anxiety-related symptoms and a greater proportion of people who answered affirmatively to the two questions we use as a lifetime screen for depression [30]. In addition, there were differences in employment status, owner-occupier of an accommodation, perception of safety inside-outside the home and experiences of discrimination. Further details of trial participants are given by Bellon et al. [13].

Table 1 presents the unadjusted mean costs and effects for the intervention and control groups.

### Cost-effectiveness analyses

Table 2 shows the adjusted means and the ICERs.

**Table 2:** Incremental cost-effectiveness ratio (ICER)-adjusted analysis: main scenarios

Effect	Ajdusted^a^ mean difference (95% bootstrapped CI) and ICER
Main Outcome	
Societal perspective	
Incremental cost	–16.38(–615 to 503)
Incremental QALY	0.02(–0.00 to 0.04)
ICER (cost per QALY gained)	Dominant
National Health perspective	
Incremental cost	23.88 (–149 to 215)
Incremental QALY	0.02 (–0.00 to 0.04)
ICER (cost per QALY gained)	€1327/QALY
Secondary outcomes	
Societal perspective	
Incremental cost	–16.38(–615 to 503)
Incremental QALY-VAS	0.02 (0.01 to 0.03)
ICER (cost per QALY-VAS gained)	Dominant
National Health perspective	
Incremental cost	23.88 (–149 to 215)
Incremental QALY-VAS	0.02(0.01 to 0.03)
ICER (cost per QALY-VAS gained)	€1085/QALY

^a^All the analyses have been adjusted by baseline variables: employment status, owner/occupier of an accommodation, perception of safety inside/outside the home, anxiety disorder, experiences of discrimination,city, in addition to the risk of depression and the respective baseline value (i.e. costs or QALYs)

**Fig. 1:**
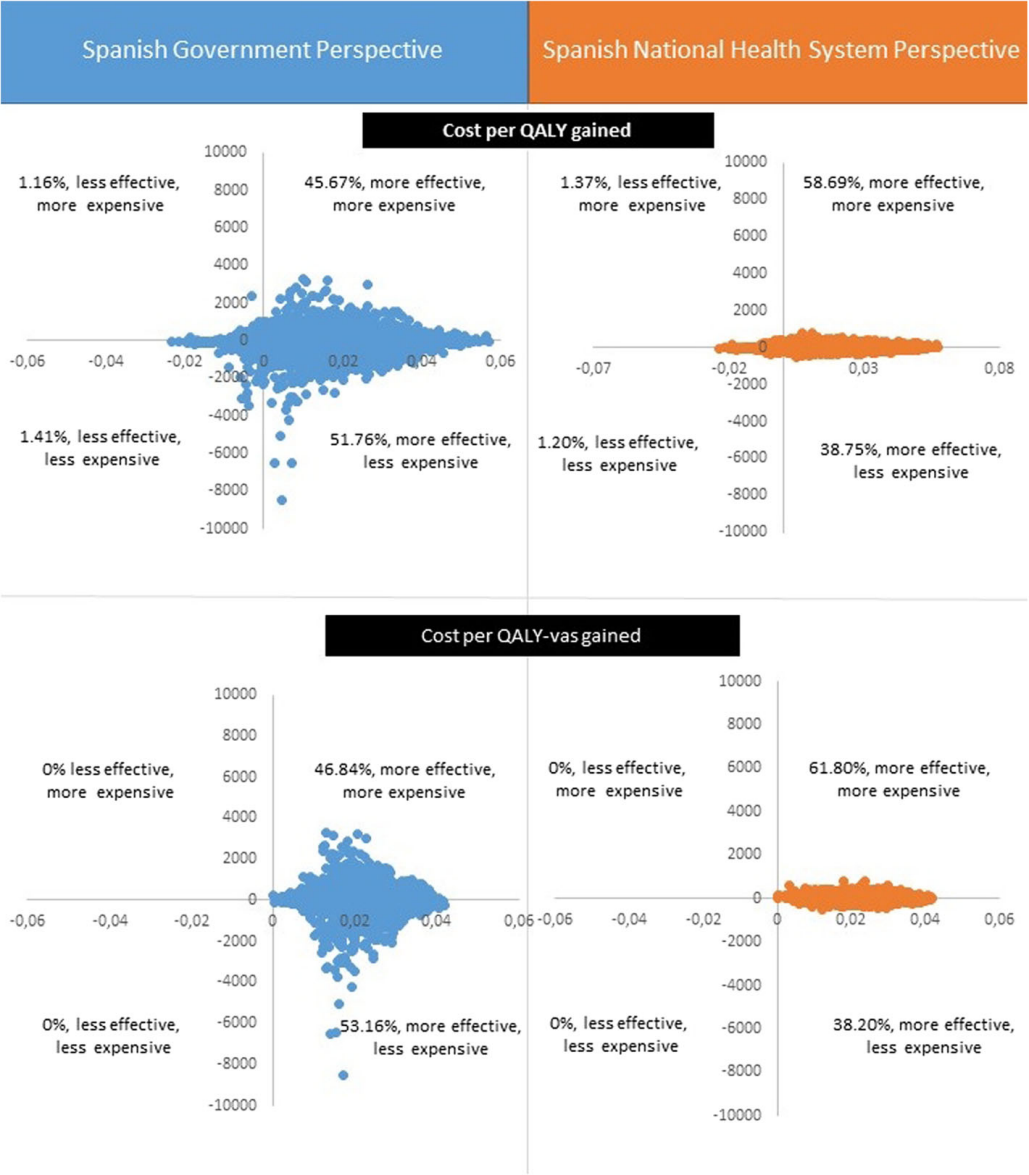
Cost-effectiveness planes

From a societal perspective the new intervention is dominant, as the increment in cost is negative and the effects are positive. However, although most (97.4%) of the incremental effects were plotted in the Eastern quadrants (new intervention more effective), the level of uncertainty related to the costs is quite high, with half of them in the Northern quadrants (new intervention more expensive) and the other half in the Southern quadrants (new intervention less expensive). These results are depicted in Fig. 1, left column.

**Fig. 2:**
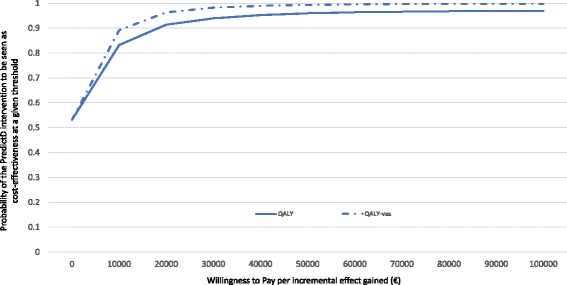
Cost-effectiveness acceptability curves (CEACs): societal perspective

The acceptability curve is shown in Fig. 2. At the €30,000 per QALY ($32,058,£25,704) threshold the probability that the PredictD intervention would be seen as cost-effective was 94%. This probability increased to 98% when considering the effect in terms of the QALYs-VAS. However, these values decreased to 83% and 89%, respectively, when a threshold of €10,000 ($10,686, £8568) was used.

**Fig. 3:**
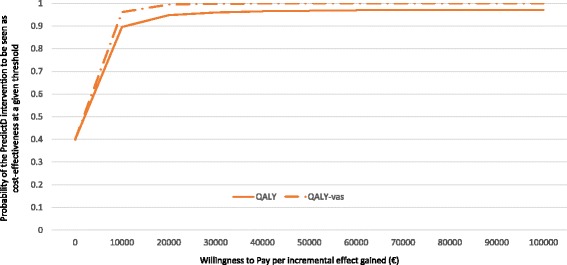
Cost-effectiveness acceptability curves (CEACs): National Health System perspective

From a National Health System perspective, the incremental cost for QALYs gained was €1326. The cost for QALYs-VAS was €1085.45. Similarly as shown for the societal perspective, although most of the incremental effects were also plotted in the Eastern quadrants, the level of uncertainty related to the costs was quite high (Fig. 1, right column). Figure 3 depicts the acceptability curve from a National Health System perspective. Similarly, at the €30,000 per QALY threshold ($32,058,£25,704), the probability that the PredictD intervention is cost-effective was 96%, increasing to almost 100% when the effect was measured using QALYs-VAS. These values decreased to 89% and 96% when a threshold of €10,000 ($10,686, £8568) was used.

### Sensitivity analysis

Table 3 summarizes the sensitivity analysis. The scenario that only considered the costs directly related to primary and mental health care was the best. The worst scenario was the one where the costs were doubled. However, the values were quite similar.

**Table 3:** Sensitivity analysis

Sensitivity analysis^a^	Adjusted mean difference (95% CI ) and ICER	Incremental net benefit: probability intervention is seen as effective with a threshold of:				
		€0	€10,000	€30,000	€50,000	€100,000
A. Perspective						
Costs only related to primary cre and mental health						
Incremental cost	€–43.61 (–186 to 10)					
Incremental QALY	0.02(–0.00 to 0.04)					
ICER (cost per QALY gained)	Dominant	0.9250	0.9852	0.9808	0.9784	0.9765
All the costs						
Incremental cost	€–10.32 (–253 to 281)					
Incremental QALY	0.02(–0.00 to 0.04)					
ICER (cost per QALY gained)	Dominant	0.537	0.8989	0.9659	0.9724	0.9751
B. Discount rate (societal perspective)						
Discount rate: 0%						
Incremental cost	€–16.19 (–612 to 500)					
Incremental QALY	0.02 (0.00 to 0.04)					
ICER (cost per QALY gained)	Dominant	0.5309	0.8347	0.9413	0.9606	0.9710
Discount rate: 6%						
Incremental cost	€–16.51 (–618 to 505)					
Incremental QALY	0.02(–0.00 to 0.04)					
ICER (cost per QALY gained)	Dominant	0.5316	0.8301	0.93855	0.9584	0.9694
C. Unit costs (societal perspective)						
Cost doubled						
Incremental cost	€–32.76 (–1231 to 1006)					
Incremental QALY (social tariffs)	0.02(–0.00 to 0.04)					
ICER (cost per QALY gained)	Dominant	0.5316	0.734	0.8874	0.9301	0.9594
Costs halved						
Incremental cost	€–8.19 (–308 to 252)					
Incremental QALY (social tariffs)	0.02(–0.00 to 0.04)					
ICER (cost per QALY gained)	Dominant	0.5316	0.9140	0.9635	0.9699	0.9734
D. Analytical strategy						
Seemingly unrelated equations (societal perspective)						
Incremental cost	€–38 (–251 to 174)					
Incremental QALY (social tariffs)	0.02(–0.00 to 0.04)					
ICER (cost per QALY gained)	Dominant	0.6383	0 .9307	0.9711	0.9750	0.9767
Completers analysis (societal perspective)^b^						
Incremental cost	€–14.46 (–1217 to 1456)					
Incremental QALY (social tariffs)	0.02 (0.01 to 0.04)					
ICER (cost per QALY gained)	Dominant	0.48	0.761	0.888	0.923	0.962
Models adjusted only by costs or QALYs at baseline (societal perspective)						
Incremental cost	€118.52 (–909€ to 1497)					
Incremental QALY (social tariffs)	0.02 (0.01 to 0.04)					
ICER (cost per QALY gained)	€5644/QALY	0.3864	0.7165	0.8727	0.9218	0.9606

^a^All the analyses have been adjusted by the following baseline variables: employment status, owner/occupier of an accommodation, perception of safety inside/outside the home, anxiety disorder, experiences of discrimination,city (unless something elsewas stated), in addition to the risk of depression and the respective baseline value (i.e. costs or QALYs)

^b^Completers analyses were weighted using inverse probability weighting

## Discussion

### Summary

Over the 18-month evaluation period, the PredictD intervention was found to be efficient. The cost-effectiveness advantage arises from the finding that the PredictD intervention increases quality of life while not significantly increasing overall costs. The sensitivity analyses confirm the robustness of the results.

### Strengths and limitations

This is the first economic evaluation nested in a randomized trial to evaluate the effectiveness of universal prevention of depression in adults implemented by GPs. Major strengths include the large sample (more than 3000 primary care attendees) and a follow-up time of 18 months, which is longer than that for most depression prevention trials.

Nonetheless, the results of this study should be considered with the following limitations in mind. First, due to the recruitment procedure, our study may have under-represented patients who attend infrequently [31]; however, frequent attenders are more at risk of major depression [32] and are most in need of prevention. Second, intervention and control groups were unbalanced on some individual variables, so that participants had a higher risk of depression in the intervention group. This is not unusual in cluster randomized control trials, where an imbalance in the characteristics of participants can creep in because randomization occurs at the level of centre [33]. To solve that, we have adjusted the results for the unbalanced variables. Third, the patients were not blind to the intervention. They may have modified their responses to satisfy the researchers/GPs. Fourth, the information on use of services was collected by means of self-report. Some bias in recall may be expected, although it is quite likely that this bias was equally distributed between the intervention and control groups. In addition, we have not taken into account informal care-related costs and costs from general medication. As depression has an impact on physical health, it is possible that this has been affected, making the costs associated with depression possibly higher than we have calculated in our study. On the other hand, the cost associated with the training of the GP was translated to the patient level by dividing by the number of participants in the trial and not by the total number of patients, which would be more appropriate in real practice. Consequently, the costs associated with the intervention in real practice would be even lower. Fifth, in our study, only 32.4% and 36.6% of patients, in the control and the intervention group, respectively, answered yes to the two questions of lifetime screen for depression (except in the 6 months prior to the baseline interview, in which no patient suffered major depression according to the CIDI) [13]. The predictive positive value of responding yes to these two questions is 18% [30],and in our study the proportion of patients who truly suffered a first episode of depression before recruitment was 5.8% and 6.6% in the control and the intervention group, respectively. Therefore, from this point of view, our study is largely based (approximately 94% of participants) on primary prevention of the onset of depression (first episode). Lastly, the generalizability of our findings may be limited because costs associated with primary care processes in Spain are less costly than in other Western countries, due to the fact that GPs are salaried [17]. However, in these other countries, such as the USA, the cost-effectiveness threshold is also higher.

### Comparison with existing literature

To the best of our knowledge, there are only two economic evaluations focused on the prevention of depression that can be compared with ours. Hunter et al. [34] carried out an economic modelling study based on the PredictD risk algorithm concluding that identifying non-depressed general practice attendees at highrisk of depression using the algorithm PredictD and providing them with a psychosocial preventive programme was potentially more cost-effective than the current practice. At a threshold of £25,000 (€30,000,$31,200 ) per QALY the probability of being cost-effective was around 70%. Our analysis showed that the probability of being cost-effective at this threshold is even higher.

Similarly, Van den Berg et al. [35] built up a model to estimate the cost-effectiveness of preventing depression in people with subthreshold symptoms of depression opportunistically approached in primary care practices. The intervention consisted of a self-help manual with instructions on cognitivebehaviour self-help in mood management plus up to six short telephone calls to support the participants while working through the manual. Given a willingness to pay of €30,000 ($32,058,£25,704) per disability-adjusted life year (DALY), the probability that the intervention was cost-effective was around 80%. Again, our intervention had a higher probability (96%) of being cost-effective at the same threshold. It may be hypothesized that participants in our trial benefited from a tailored face-to-face intervention as well as from the opportunistic reviews when they consulted their GPs related to other issues.

However, in both economic studies [34, 35] strategies for preventing depression in high-risk patients were evaluated (selective or indicated prevention), whereas in our study we followed a universal and personalized prevention implemented by GPs.

### Implications for practice

This intervention differs from other interventions to prevent depression because it is tailored to each patient’s individual level and profile risk. This approach parallels primary prevention of cardiovascular diseases in primary care, although the risk factors involved and their management are different. Indeed, our intervention did not increase costs because it was embedded into the day-to-day practice. Our study showed that universal prevention of depression in adults, using the PredictD intervention and implemented by GPs, has a high probability of being cost-effective compared to usual care. However, with our study we cannot know if this type of universal prevention would be more cost-effective than other types of primary prevention (selective or indicated). Further trials comparing these types of prevention and different frequencies of risk evaluation are need. GPs generally perceive that they do not have enough time to perform preventive activities, and they may agree to do so only for high-risk patients. Another possibility would be to provide universal prevention to patients by mean of a scalable and cheap strategy (e.g. through apps and smartphones), reserving the intervention of GPs only in cases of high risk of depression. Our research team is currently conducting a new trial with the latter strategy (the e-PredictD study).

## Conclusions

The PredictD intervention is likely to be perceived as cost-effective, from both a societal and National Health System perspective, compared to usual care.There is, therefore, both a clinical and an economic case for supporting the implementation of this intervention, which is based on the level and profile of risk for depression, in primary care practices. However, this intervention should be further developed and evaluated in other countries.

## Abbreviations

CCRT: Cluster controlled randomized trial
CEAC: Cost-effectiveness acceptability curve
CSRI: Client Service Receipt Inventory
DALY: Disability-adjusted life year
GLM: Generalized linear model
GP: General practitioner
ICC: Intraclasscorrelation coefficient
ICER: Incremental cost-effectiveness ratio
NICE: National Institute for Health and Care Excellence
QALY: Quality-adjusted life year
SUR: Seemingly unrelated regression
VAS: Visual analogue scale
WTP: Willingness to pay
